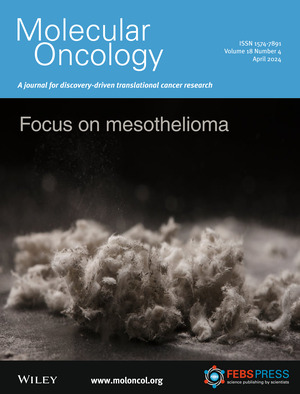# Issue Information

**DOI:** 10.1002/1878-0261.13458

**Published:** 2024-04-04

## Abstract

The cover image depicts asbestos dust, exposure to which is a major factor in the development of mesothelioma. It aligns with the central theme of this issue, which delves into various aspects of mesothelioma, alongside articles on sarcomas, rare cancers, and melanoma. Additionally, this issue features a policy article penned by our editorial board members [pp. 785–792].